# Abstract of the Proceedings of the Medical Association of the State of Georgia

**Published:** 1859-05

**Authors:** 


### EDITORIAL AND MISCELLANEOUS.

### ABSTRACT OF THE PROCEEDINGS OF THE MEDICAL ASSOCIATION OF THE STATE OF GEORGIA.

The Medical Society of the State of Georgia, convened in the city of Atlanta, on Wednesday morning, the 13th of April, and was called to order by the President, Dr. J. P. Logan. The Secretary being absent, Dr. Meire, of Madison, was elected Secretary, *pro tem.*

The number in attendance was very large. After a large addition of new members, and some other business, the Society adjourned until 3 o'clock, P. M.

The election of officers being in order, Dr. Colley, of Munroe, Walton county, was selected for President, Dr. R. A. T. Ridley, of LaGrange, 1st Vice-President, Dr. Hayden Coe, of Atlanta, 2d Vice-President, Dr. A. G. Thomas, of Atlanta, Secretary, Drs. Campbell, of Augusta, Battey, of Rome, and Alexander, of Atlanta, were appointed a Committee to conduct the President elect, to the Chair. Before vacating, Dr. Logan, the retiring President, delivered the following address, which was endorsed, and ordered to be published:

*Gentlemen of the Medical Society of the State of Georgia—*

In retiring from the honorable position assigned me, by the partiality of your body at its last annual session, held in Madison in April 1858, I would do great injustice to myself, and fail to be properly responsive to the obligations imposed, did I not take this brief opportunity to express the high sense which I entertain of the honor conferred upon me, and the humble, but earnest zeal, with which I have sought to sustain the trust confided to me.

At the close, therefore, of my annual service, and in accordance with the usage of such associations, I trust I may be indulged in a few reflections which I regard as not inappropriate to the present occasion. I congratulate you, gentlemen, upon the full attendance which marks our present session. The profession from the various and wide extended portions of this great commonwealth, is more largely represented upon this floor, than it has been upon any similar occasion for years past; and I would fain hope, from the unusual interest which you have manifested by your attendance upon this occasion, that it is the dawn of a brighter era in the history of the Medical Society of Georgia.

Almost from the seaboard to the mountains, from the hills and the vallies, and from the borders of surrounding States, from the north and the south, from the east and the west, you have come, gentlemen, to the railroad city of the south, where, in a few years, a large and busy population, actively engaged in performing their part in the great drama of civilization, has taken the place of the wilderness and the savage.

Permit me to hope, gentlemen, that your organization—the representative of a science and an art, fraught with so many of the blessings of that civilization—may receive a new impulse from the striking illustration which is here furnished of the rapid progress of an age, which will constitute an era in the history of the human race.

The *place* and the *occasion* are appropriate, from which to derive a new impetus which shall inspire us with a new zeal and a more active and determined purpose, to keep pace with the rapid progress, which is so signally characteristic of this age in almost every department of science and art.

We have come up this day from the extreme borders of this great and sovereign State, to fraternize with each other in a fresh act of homage to our venerated calling, and to commune with and strengthen each other by new vows of devotion to the claims and advancement of a profession, which has for its end, the noblest earthly object, the relief of suffering humanity. We have come up to show and acknowledge our allegiance to the honored organization, whose wisdom and authority we recognize, as the *supreme* law to every true medical man within the broad limits of the noble commonwealth to which we, this day, declare our devotion, and recognizing no "higher law" in a political point of view, (except, so far as power may have been derived from direct delegation,) than that found in the government of our own sovereign State; so we know no absolute controlling power, in a medical point of view, beyond the limits of this organization; though we shall ever feel inclined to respect the moral prestige and influence of all high professional bodies, having for their end the same high object with ourselves—the advancement of science, and the elevation of our profession.

The annual meetings, then, of your society, gentlemen, are occasions of no ordinary or trivial character; an investigation into our scientific and ethical position, pressing with full force upon us; with none the less force certainly, because we have united for the general interests and advancement of medical science, with the profession of a vast continent, in what is called the American Medical Association—an organization confined within its legitimate sphere, of vast and growing importance; perverted, from what should be its true purposes and objects, and entering upon a crusade for the correction of abuses, whether real or imagined, in the ramifications of medical affairs within the jurisdiction of State organizations, destined to a speedy dissolution.

But, gentlemen, it is the precise epoch at which we have arrived in the world's history, and in our own history, which imparts a peculiar interest and importance to medical investigation. The accumulated knowledge of the past, constitutes the lofty elevation from which a wider panorama opens to succeeding generations. We hesitate not to affirm, that this is not the less true in reference to the science of medicine, than in regard to any other branch of human knowledge.

Notwithstanding the efforts of those who adopt as a profession or follow in the wake of some one or another of the protean forms of modern empiricism and Medical folly, combined with the slanders and detractions of some in the profession who keep up the stereotyped cry of the defects of American Medical education, and the inferiority of American Medical men—their own professional character the result of this system. Notwithstanding, I say, the existence of those who either do not know, or, forget, the direction of the march and progress of civilization and are ever looking backwards to the decaying institutions of Europe for models worthy of imitation and praise, and who can see no good in the present or hope in the future, it is yet true that Medical Science is progressive, and to no small extent is this true, as the result of the labors and contributions of your own countrymen. Without multiplying examples (which it would not be difficult to do, if the time and occasion permitted it,) it is only necessary to refer to the recent brilliant achievements of Sims and Bozeman (not only Americans but from your own South and both from your adjoining State Alabama,) developed in the case of the former into that noble institution, the Woman's Hospital, New York, and in the case of the latter into a triumphal tour through Europe compelling the acknowledgment at last, that European may learn something from American Medical men. Yes, gentlemen, from your very homes, as it were, from your very midst have arisen men who will be hailed as the greatest benefactors by every intelligent female, until the latest generation to whom the sad calamity shall befall, for the relief of which they have accomplished so much.

I do not consider it inappropriate to the present occasion, and I cannot forbear to declare, gentlemen, that it is only lately that the meed of praise and reluctant adoption has been awarded an American idea in the hospitals of Paris. We are informed that Dr. Suckly, of New York, late assistant surgeon in the United States Army, and author of a valuable publication on the birds and animals of Oregon Territory, has carried to Paris and presented to M. Nelaton the distinguished Professor of Clinical Surgery at the Hospital of the Faculty, an apparatus for fractures, now in *common use*, in the hospitals of the large American cities, the successful (and to him astonishing) results, of which, in the New York and Philadelphia Hospitals, he utterly refused to acknowledge; he, however, politely placed a fractured leg at Dr. Suckley's disposition, who applied the American apparatus, and the result was so remarkable as to perfectly-convert M. Nelaton to the American method. In the meantime Dr. Suckley obtained from Gurdon Buck, an authenticated copy of the results of the treatment of fractures in the the city hospital New York, with this apparatus during a series of years, and presented the report to Nelaton, and in view of the extraordinary results there obtained, the eminent surgeon of the Faculty Hospital, whose influence is said to be more powerful than that of any other man in the Faculty of Paris, was obliged to yield precedence to the "American System," and since this time has used none other in his Hospital. And thus, gentlemen, examples might be multiplied, if it were necessary, to sustain the position, that it is far more true now than it was at a former period, when Caldwell, of Kentucky, and Paine, of New York, came to the defence of the American medical profession, and established the fact, that American medicine has nothing to fear from a just comparison with that of the Old World.

While I would urge upon you by every consideration connected with the relief of suffering humanity, and the solemn responsibility connected with the assumption of the office of physician, the great importance of using every means in your power to advance our science, and especially your own individual skill in the treatment of disease, I shall never unite with those in the profession who vilify and abuse American medical men, and the "American *system* of medical education," so long as I have the most reliable authority for stating, and so long as I believe that in practical knowledge and skill in the treatment of the diseases belonging to their country, the regular physicians of the United States are superior to any medical men upon the globe, so long as the large and valuable contributions to medical science contained in the annual volume of the transactions of the American Medical Association, and the smaller yet creditable additions made through the transactions of the various State Medical Societies, and the constant and almost innumerable contributions to the medical journals of the country from the thousands of colaborators in the practical walks of medicine, scattered throughout this vast Union, convince me that at this day our science is making more rapid advances in America than in any part of the earth. No, gentlemen, yours is not a degraded calling, as our pseudo "reformers" would make you believe, and the assaults of traitors (to their obligations to respect and honor their profession) within your ranks, or renegades from regular medicine, or of the horde of quacks, are only the buzzings of insects around the ascending bird of heaven.

Holding these views, and in my judgment there being a very unnecessary excitement upon the subject of medical education, and believing that it is a subject that will take care of itself, and like other departments of active life, under our free and liberal institutions, and under the stimulus of competition, will always furnish an amount of skill equal to the demand—contending as I do that in connection with the Medical Colleges, the Societies of each State are the rightful guardians of their own local medical affairs, and are the only professional bodies that have any jurisdiction whatever over this subject, which is agitating the medical mind to a considerable extent at this particular period, and which has continually disturbed the harmony of the American Medical Association from its very commencement, I have thought it not improper to make an allusion to it. Allow me, gentlemen, to admit that there are unworthy and unqualified men in our ranks, not to a greater extent, however, than in any other calling of your country, the entrance of whom into the profession will not, in my judgment, ever be prevented; but if, by any method, certainly none holds out the remotest prospect of success, but *the* (Wood's Report,) *separation of the teaching and licensing power.* Certain it is that the harmony and welfare of the profession requires a settlement of this truly "vexed question," and holding the views which I do, I have considered myself at liberty to take a passing glance at the subject.

But, gentlemen, I have already stated that I am not among the *croakers* either in reference to the science or the art. I

have already intimated that in my judgment, medicine was keeping pace with the rapid progress of every other department of science or art. Life is being prolonged; the duration of disease is being shortened, and incurable diseases have regularly and steadily diminished; the resources of our art have continually increased, its sphere has continually widened, and with all its clogs and embarrassments, it assumes higher ground to-day than at any former period of the world's history; and gentlemen, this is just what ought to be expected from the spirit of the age—*truth* has nothing to fear from, but *courts* free investigation, and is established by free inquiry. Improvement is only made by encouraging freedom of thought—making innovations upon the past, repudiating obsolete and unreasonable dogmas, however venerable for years, and yielding to the claims of truth and the light of experience.

But then, gentlemen, if the healing art occupies the high position to which I would assign it, the office of physician is one of serious and responsible character—it is one of lofty and imposing claims and influences, and when properly appreciated, and its duties faithfully discharged, is honorable in itself and a blessing to the world. But its very importance and capacity for good, makes it also, an instrument for evil, if not properly estimated, or if unfaithfully administered. It is hoped that each one of us will be responsive to the claims of our high vocation, and by a faithful discharge of duty, diffuse rich blessings upon our fellow men, and merit the respect and confidence of the world; but ever recollect, that in our most successful efforts, we are merely instruments in the hands of divinity, and that it is "God who healeth our diseases, and redeemeth our lives from destruction." To enforce respect from the world, however, gentlemen, we must ever respect ourselves and cherish that spirit of harmony and peace which should ever belong to gentlemen and members of an ancient, noble and philanthropic profession.

Let us ever remember that those who malign each other, must ever expect the world to depreciate them; and that those who voluntarily degrade themselves, deserve nothing less than degradation. Let this happy "reunion" provoke us all to love and good works to each other, that the world may cease to taunt us with the slander, that selfishness and envy are the besetting sins of the medical profession. Let our lives be guided by lofty principles, and be directed to salutary ends, that whether appreciated or not, we may aid, in dispelling the darkness and gloom, that disease and death ever hangs over our earth, and that we may aid in diffusing a grateful warmth and light, which will at once invigorate and refresh.

And, now, gentlemen, as the organ of the profession of this city, allow me to express the hope, that this meeting may prove a gala day to the brotherhood who have assembled in our midst—a day of relief, and of joy, and of "good cheer," to those whose life is one of self-sacrifice, labor and toil. Allow me, in their name, to extend to your entire body, specially and individually, a most cordial welcome to our city, and the hospitality with which I am authorized to say, they desire to greet you, and which they hope you will accept and enjoy.

Gentlemen, it is no ordinary pleasure which I, this day, experience, to see so many of the medical men of Georgia, assembled in council. I am most happy to be with you—and now in retiring from this chair, allow me again, to thank you for the honor conferred upon me, and to express the earnest hope that all your deliberations may tend to the good of our whole profession, and that the results may redound to the honor and advancement of medical science, and to the prosperity of our calling. And, finally, to my excellent and honored successor, I cheerfully resign, in accordance with ancient and approved usage, the chair with which you have honored me, trusting, that when in the order of providence we may next meet, he may exhibit the standard of our profession, floating higher and broader, before the gaze of the world.

The following gentlemen were appointed delegates to the American Medical Association:

Drs. West and Sullivan, of Savannah, Doughty and Robt. Campbell, of Augusta, Nottingham and Boone, of Macon, Means, of Oxford, Alexander, of Atlanta, Stanford and Flewellen, of Columbus, McCluskey and Carlton, of Athens, Roach, of Pulaski, Stevens, of Albany, Hillyer and Battey, of Rome, Danielly, of Merriwether, Burney, of Forsyth, McAfee, of Dalton, Ridley, of LaGrange, Banks, of Pike, Brown, of Cumming, Miere, of Madison.

The name of the Body was changed from Medical Society of the State of Georgia, to Medical Association of the State of Georgia.

There were quite a number of interesting and valuable Essays presented to the Association; among the number, one from Dr. Doughty, of Augusta, on Climates best adapted to Consumptives; one from Dr. Robt. Campbell, of Augusta, on Quinine, in which he attempts to show that "its therapeutical action is expended solely upon the *middle* or *fibrinous coat of the blood vessels*, by which interpretation alone, its phenomena are satisfactorily explicable." Two Reports were presented from Dr. Dugas, of Augusta—one on a Fracture of the Neck of the Scapula, the other on Aneurism, occurring in the Gluteal region. Dr. Jos. Jones, of Augusta, presented an Essay on the Changes of the Blood in Malarial Fevers. Dr. H. F. Campbell, of Augusta, presented an Abstract of a Report of fifteen cases of Lithotomy with the Calculi accompanying. Dr. T. H. Dean, of Conyers, presented an Essay on Puerperal Fever. Dr. A. G. Thomas, of Atlanta, presented an Essay on Quackery and its Cure. Dr. Robt. Battey, of Rome, presented a Report of a case of Vesico Vaginal Fistula. Dr. Hayden Coe, of Atlanta, presented an Essay on the Pathology of Phlegmasia Dolens.

Upon motion of Dr. Robt. Battey of Rome, it was

*Resolved*, that a Committee of three be appointed by the Chair, to report at our next meeting, upon the evidences of advancement in Medical Science, as exhibited in the literary productions of the medical men of Georgia.

The Committee appointed for this purpose, were Doctors

Logan, of Atlanta, Battey, of Rome, and Sullivan, of Savannah.

The plan of publishing the transactions of the Association, was altered—allowing each Essayist to select any Medical Journal within the State, in which to publish his contributions.

Dr. H. W. D. Ford, of Augusta, was elected Orator for the next annual meeting; Dr. Banks, of Pike, Alternate. The city of Rome was selected as the next place of meeting; and Drs. Battey, T. J. Word, Miller, Hillyer and R. C. Word, were appointed a Committee of Arrangements for the next meeting.

We furnish above, a very meagre and brief abstract of the proceedings of the Medical Association of the State of Georgia, at its last Annual Meeting, held in Atlanta, on the 13th and 14th of April, 1859.

The attendance was unusually large, and the contributions much more extensive and valuable than usual, and perhaps unprecedented, and will (in detail,) compare favorably in value, if not in number, with those which are furnished to the American Medical Association. We shall furnish our readers with the official report of the meeting in the next number of our journal, and in succeeding numbers will publish the whole of the Essays of those gentlemen who furnish them for that purpose, in accordance with the discretion granted them by the Society; and in addition, we propose to publish abstracts of those furnished to other journals.

The late meeting of the Association here, was, we believe, considered by all who attended, as a most pleasant "reunion," and as the inauguration of a new era in the history of the organization.

